# Shared and independent functions of aPKCλ and Par3 in skin tumorigenesis

**DOI:** 10.1038/s41388-018-0313-1

**Published:** 2018-05-23

**Authors:** Susanne Vorhagen, Dominik Kleefisch, Oana-Diana Persa, Annika Graband, Alexandra Schwickert, Michael Saynisch, Michael Leitges, Carien M. Niessen, Sandra Iden

**Affiliations:** 1 Department of Dermatology, University of Cologne, Köln, Germany; 2 Cologne Excellence Cluster on Cellular Stress Responses in Aging-Associated Diseases (CECAD), Köln, Germany; 3 Center for Molecular Medicine Cologne (CMMC), University of Cologne, Köln, Germany; 4 Biotechnology Centre of Oslo, University of Oslo, 0316 Oslo, Norway

## Abstract

The polarity proteins Par3 and aPKC are key regulators of processes altered in cancer. Par3/aPKC are thought to dynamically interact with Par6 but increasing evidence suggests that aPKC and Par3 also exert complex-independent functions. Whereas aPKCλ serves as tumor promotor, Par3 can either promote or suppress tumorigenesis. Here we asked whether and how Par3 and aPKCλ genetically interact to control two-stage skin carcinogenesis. Epidermal loss of Par3, aPKCλ, or both, strongly reduced tumor multiplicity and increased latency but inhibited invasion to similar extents, indicating that Par3 and aPKCλ function as a complex to promote tumorigenesis. Molecularly, Par3/aPKCλ cooperate to promote Akt, ERK and NF-κB signaling during tumor initiation to sustain growth, whereas aPKCλ dominates in promoting survival. In the inflammatory tumorigenesis phase Par3/aPKCλ cooperate to drive Stat3 activation and hyperproliferation. Unexpectedly, the reduced inflammatory signaling did not alter carcinogen-induced immune cell numbers but reduced IL-4 Receptor-positive stromal macrophage numbers in all mutant mice, suggesting that epidermal aPKCλ and Par3 promote a tumor-permissive environment. Importantly, aPKCλ also serves a distinct, carcinogen-independent role in controlling skin immune cell homeostasis. Collectively, our data demonstrates that Par3 and aPKCλ cooperate to promote skin tumor initiation and progression, likely through sustaining growth, survival, and inflammatory signaling.

## Introduction

One hallmark of carcinomas is the loss of cell polarity associated with altered epithelial organization and cell behavior. However, whether and how these alterations in epithelial organization drive malignant transformation and progression remains poorly understood. The cell polarity proteins Par3, Par6, and aPKC form the aPKC/Par complex and are key regulators of epithelial apico-basal polarity, migration, cell fate, and inflammation [1]. Most of the loss-of-function data indicate that these proteins exert their function as part of this complex, albeit that in mammals a direct genetic comparison has not been done. Several findings also suggest that the complex is subject to highly dynamic regulation and that each of these proteins may serve complex-independent functions. For example, aPKC has other interaction partners that compete with either Par6 or Par3 binding, and Par3 dynamically interacts with aPKC/Par6 in different functional settings [2, 3]. Recent evidence suggests that in breast cancer cells Par3 restricts aPKC function and downstream inflammatory signaling [4, 5]. Finally, several observations indicate potentially independent roles for Par3 and aPKC, e.g., in DNA repair, inflammation, proliferation and epithelial structure [6–9]. Complicating matters further, for each protein several isoforms exist, encoded by different genes: aPKCζ, aPKCλ, as well as Par3A and Par3B.

In human cancer, aPKCλ is often overexpressed, whereas for Par3 both increased and decreased expression was observed [10–14], suggesting that these proteins have opposite actions also in human malignancies. Different mouse tumor models confirmed that aPKCλ serves as a tumor promoter [15] whereas depending on the tumor settings Par3 indeed can serve both tumor-promoter and suppressor functions [4, 13, 15].

We previously identified Par3 as a promoter of 7,12-Dimethylbenz(a)anthracene (DMBA)-induced epidermal skin cancer. Our in vitro data indicated that Par3 controls mutant Ras-driven proliferation through aPKC [13]. Par3 and aPKCλ, the predominant aPKC isoform expressed in skin [16], are also major regulators of epidermal homeostasis. Loss of either protein resulted in hair follicle stem cell loss and increased differentiation, whereas these two proteins served opposite functions in regulating spindle orientation [16, 17], a mechanism implicated in epidermal stem/progenitor fate decisions [18, 19], suggesting both shared and distinct functions. Consequently, this tissue thus provides an excellent model system to directly address whether and how aPKCλ and Par3 act in concert or independently in driving skin carcinogenesis. We therefore employed genetic loss-of-function mouse models and examined how loss of aPKCλ, Par3, or both, affect growth, apoptosis and inflammation at different stages of skin tumorigenesis.

## Results and discussion

### Par3 and aPKCλ cooperate in skin tumorigenesis

**Fig. 1:**
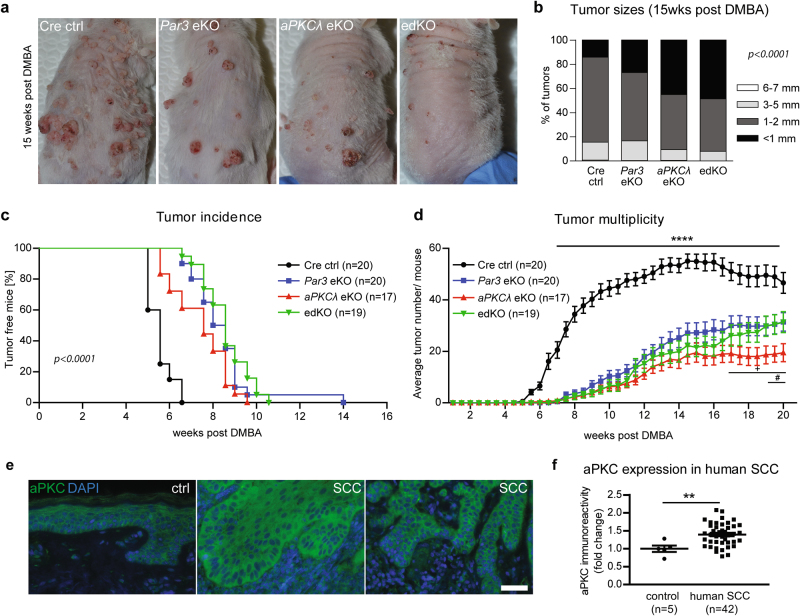
Par3 and aPKCλ cooperate in skin tumorigenesis. Cre^+^ controls (K14^Cre/+^), epidermis-specific Par3A (*Par3*eKO: K14^Cre/+^; *Par3*^fl/fl^), *aPKCλ* (*aPKCλ*eKO: K14^Cre/+^; *aPKCλ*^fl/fl^) and double-knockout mice (edKO: K14^Cre/+^; *Par3*^fl/fl^; *aPKCλ*^fl/fl^) were generated in a mixed FVBN/ C57BL/6N F2 background (K14-Cre [38]; *Par3F* [39]; *aPKCλF* [40]). Two independent two-stage skin carcinogenesis experiments were performed via long-term topical DMBA/TPA treatments as previously described [13]. 20 Cre ctrls, 20 *Par3*eKOs, 17 *aPKCλ*eKOs, and 19 edKO mice were examined biweekly and tumor numbers and sizes were measured. Biometric analysis considering effect size, variation, type 1 and type 2 errors were performed to determine required group sizes that enable appropriate power. **a** Macroscopic tumor phenotypes of long-term DMBA/TPA-treated mice at 15 weeks after DMBA treatment. **b** Quantification of tumor sizes at 15 weeks post-DMBA treatment (*p* < 0.0001; Chi square test with 4 × 4 contingency table). **c** Tumor incidence upon loss of Par3 and aPKCλ in response to long-term DMBA/TPA treatment (*p* < 0.0001; survival curves/ log-rank Mantel-Cox test; also *p* < (control vs. each of the mutant lines) <0.0001 by “two-at-a-time” comparison followed by Bonferroni correction, and non-significant between individual mutant lines). **d** Tumor multiplicity upon loss of Par3 and aPKCλ in response to long-term DMBA/TPA treatment (mean ± SEM; 2way-ANOVA/Tukey’s post-hoc test; ****p(control vs. each of the mutant lines) <0.0001, +: p(*aPKCλ*eKO vs. *Par3*eKO) <0.05, #: p(*aPKCλ*eKO vs. *Par3*eKO and edKO) <0.05. Experiments were approved by and annually reported to the governmental authorities (“Landesamt für Natur, Umwelt und Verbraucherschutz Nordrhein-Westfalen,” State Office North Rhine-Westphalia, Germany), and performed according to institutional guidelines and national animal welfare regulations. Sick, underdeveloped or by any other means atypical animals were excluded from the analyses. Mice of different genotypes were randomly co-housed. Males and females were used for experiments, with its ratio kept comparable between test and control groups. Mice of different litters derived from different parents were included in the analysis. The mouse IDs were unrelated to genotype, and a largely blinded analysis was performed e.g., for assessment of tumor initiation and growth in living mice, and subsequent dissection. **e** Immunofluorescence analysis as previously described [13] for atypical protein kinase C (aPKC) (sc-216, Santa Cruz Biotech.) on paraffin sections of a tissue microarray consisting of 42 samples for human squamous cell carcinoma (SCC) and 5 age-matched control skin samples (scale bar = 50 µm). The tissue microarray was constructed by collecting 2 mm tissue cores from paraffin-embedded samples and placed on host paraffin blocks using a manual tissue arrayer (MTA-1, AlphaMetrix Biotech). **f** Quantification of aPKC immunoreactivity in a tissue microarray of human SCC (*n* = 5 controls, *n* = 42 human SCCs; mean ± SEM; **p < 0.01; unpaired two-sided *t*-test). Informed consent was obtained from all donors and patients involved in this study (BioMaSOTA form, University Hospital Cologne), and sampling of human specimen was approved by the ethic’s committee of the Medical Faculty of the University of Cologne (file reference 12–163)

To investigate whether aPKCλ and Par3 cooperate or exert separate functions in tumor initiation and progression, single epidermal *Par3* (*Par3*eKO) and *aPKCλ* (*aPKCλ*eKO) as well as *Par3*/*aPKCλ*-double knockout mice (edKO) were generated using K14-Cre [13]-mediated recombination (Suppl.Fig. 1A), and subjected to DMBA/2-O-tetradecanoylphorbol-13-acetate(TPA)-mediated two-stage skin carcinogenesis regimen [20, 21]. DMBA induces mutations in a variety of genes, with predominant, activating mutations in *Ras* genes [22, 23]. In agreement, all tumors examined in this study were positive for the oncogenic *Hras* Q61L hot-spot mutation [20] (Suppl.Fig. 1B). TPA triggers hyperproliferation and inflammation, thereby promoting tumor outgrowth. Loss of aPKCλ significantly reduced DMBA/TPA-induced papilloma multiplicity and delayed tumor formation (Fig. 1a, c, d), similar to loss of Par3 (Fig. 1a, c, d) [13], identifying aPKCλ as a tumor promoter in this non-melanoma skin cancer model. Importantly, combined loss of Par3 and aPKCλ did not further reduce tumor burden or delay tumor incidence, providing genetic evidence that Par3 and aPKCλ cooperate to promote skin tumorigenesis. Only at very late stages *aPKCλ*eKO mice showed a slightly reduced but significant difference in tumor numbers compared to *Par3*eKO and edKO mice (Fig. 1d), suggesting a tumor-suppressive role for Par3. Moreover, Par3 but not aPKCλ loss promoted a significant increase in keratoacanthomas (Suppl.Fig. 1F). Together, these data confirm the previously reported dual role for Par3 in skin cancer [13]. Measurements of tumor sizes at 15 weeks post-DMBA revealed significantly smaller tumor diameters in *Par3*eKO, *aPKCλ*eKO, and edKO mice compared to controls (Fig. 1b). Interestingly, although tumor burden, incidence and size were reduced, these tumors showed an increased invasive, albeit epithelial, phenotype (Suppl.Fig.1C–E). Importantly, immunohistochemical analysis revealed a significant upregulation of aPKC in human squamous cell carcinoma, suggesting that increased aPKC levels may also promote tumorigenesis in human skin cancer (Fig. 1e, f). Thus, mammalian aPKCλ and Par3 act in concert to promote DMBA-induced skin carcinogenesis in vivo while serving an invasion-suppressive function at later stages of carcinogenesis.

### Increased apoptosis and sustained DNA damage upon DMBA treatment

**Fig. 2:**
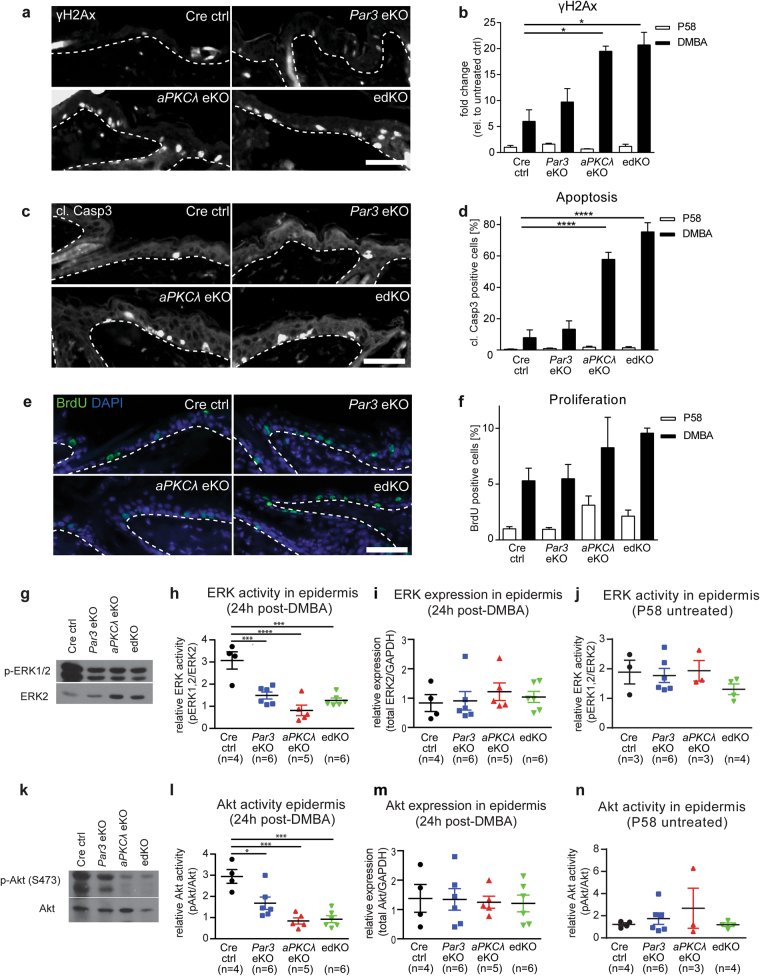
Loss of Par3/aPKCλ elicits increased apoptosis and sustained DNA damage upon DMBA treatment. For short-term DMBA treatments, mice were subjected to a single topical dose of DMBA at an age of 8 weeks (30 µg in 200 µl acetone, Sigma-Aldrich) and killed 24 h after treatment. **a** Immunofluorescence analysis for γH2Ax (Ab2893, Abcam) on paraffin sections of mice 24 h after DMBA treatment (scale bar = 50 µm) was performed as previously described [17], using 10% normal goat serum for blocking. **b** Quantification of γH2Ax-positive cell nuclei in the epidermis of DMBA treated and untreated mice at P58 (fold change to untreated Cre ctrl = 1; *n* > 8 mice/ genotype; mean + SEM; *p < 0.05; Kruskal-Wallis/Dunn’s post-hoc test). Tile-scans of entire tissue sections were generated (DM6000B; Leica Microsystems, Wetzlar, Germany) and γH2Ax-positive cell nuclei were manually counted, using DAPI as a counterstain. **c** Immunofluorescence analysis for cleaved Caspase3 (cl. Casp3) (#9664, Cell signaling) on paraffin sections of mice 24 h after DMBA treatment (scale bar = 50 µm) as previously described (2A). **d** Quantification of cl. Casp3-positive cell nuclei in the epidermis of DMBA treated and untreated mice at P58 as described in 2B (% of DAPI-positive cell nuclei; *n* > 9 mice/genotype; mean + SEM; ****p < 0.0001; one-way ANOVA/Dunnett’s post-hoc test). **e** Immunofluorescence analysis as described in (a) for BrdU (#347580, BD Bioscience) on paraffin sections of mice 24 h after DMBA treatment (scale bar = 50 µm). Nuclei were counterstained with DAPI. Mice were injected intraperitoneally using 25 µg BrdU (Calbiochem) per kg bodyweight 1 h prior to killing. **f** Quantification of BrdU-positive cells in the basal layer of DMBA treated and untreated mice at P58 as described in 2B (% of DAPI positive cell nuclei;  *n* > 4/genotype; mean + SEM, one-way ANOVA/Dunnett’s post-hoc test). **g** Example and **h** quantification of western blot analysis for ERK activity (p-ERK1/2/ ERK2) (Cell Signaling Techn., #9101 and BD Biosciences, #610104) on epidermal lysates of mice 24 h after DMBA treatment. Epidermal-dermal separation of back-skin, measurement of protein concentration, SDS-PAGE, wet transfer, and Western Blot were performed as previously described [17]. Densitometry of protein bands was performed using ImageJ (Version 1.50 g; National Institutes of Health, USA) on scanned western blots. Phosphorylated protein was normalized to total protein levels and total protein to GAPDH (Millipore, #MAB374), used as loading control (mean ± SEM; ***p < 0.001; ****p < 0.0001; one-way ANOVA/Tukey’s post-hoc test). **i** Quantification of western blot analysis for ERK expression (ERK2/GAPDH) (BD Biosciences, #610104, Millipore MAB374) on epidermal lysates of mice 24 h after DMBA treatment (mean ± SEM; one-way ANOVA/Tukey’s post-hoc test). **j** Quantification of western blot analysis for ERK activity (p-ERK1/2/ ERK2) in untreated P58 mice as described in h (mean ± SEM; one-way ANOVA/Tukey’s post-hoc test). **k** Example and **l** quantification of western blot analysis for Akt activity (pAkt (S473)/ Akt) (Cell Signaling Techn., #4060, #9272) on epidermal lysates of mice 24 h after DMBA treatment. GAPDH was used as loading control (mean ± SEM; ***p < 0.001; *p < 0.05; one-way ANOVA/Tukey’s post-hoc test). **m** Quantification of western blot analysis for Akt expression (Akt/GAPDH) (Cell Signaling Techn., #9272, Millipore MAB374) on epidermal lysates of mice 24 h after DMBA treatment (mean ± SEM; one-way ANOVA/Tukey’s post-hoc test). **n** Quantification of western blot analysis for Akt activity (p-Akt/ Akt) in untreated P58 mice as described in **l** (mean ± SEM; one-way ANOVA/Tukey’s post-hoc test). Technical assessment in terms of staining specificity, transfer efficiency for immunoblots and others was performed each time, whereby positive (where available) and negative controls were used for evaluation. For quantification of different properties (apoptosis, oncogenic signaling, BrdU incorporation, protein expression) blinding was implemented at the level of micrographs that were captured for further analyses. Additionally, automated and unbiased approached, e.g., using CellProfiler and ImageJ software, was used to obtain most objective results. See Suppl. Table 1 for antibody dilutions

A critical step for DMBA-induced tumor initiation is whether mutant keratinocytes survive to give rise to skin tumors. To decipher if loss of Par3 and/or aPKCλ affects cell survival, mice were subjected to a single topical dose of DMBA and analyzed 24 h later. Surprisingly, immunofluorescence analysis for phosphorylated histone H2Ax (γH2Ax), suggestive of sites of DNA damage, revealed a significant increase in γH2Ax-positive cell nuclei in DMBA-treated *aPKCλ*eKO and edKO compared to control mice, with a less pronounced non-significant increase in *Par3*eKO mice (Fig. 2a, b). In agreement, apoptosis, as evidenced by cleaved Caspase3 (Fig. 2c, d), and DMBA-induced p53 (Suppl.Fig. 2C–E) was significantly increased in DMBA-treated *aPKCλ*eKO and edKO mice. These data might suggest that aPKCλ plays a dominant role over Par3 in maintenance of cells harboring carcinogen-induced DNA lesions. Alternatively, as Par3 has previously been implicated in repair of γ-irradiation-induced DNA double-strand breaks [6], Par3 loss might elicit a similar effect but with different kinetics.

To investigate whether this increase in γH2Ax was accompanied by hyperproliferation, which may indicate replicative stress, short-term BrdU incorporation or PCNA staining was assessed, which revealed no significant differences between any of the genotypes at 24 h post-DMBA (Fig. 2e, f, Suppl.Fig. 2A, B). Together, these data indicate that epidermal loss of Par3 or aPKCλ results in a differential sensitivity to both DNA damage and apoptosis, which, however, cannot explain the similar tumor incidence and multiplicity seen in *Par3*eKO, *aPKCλ*eKO, and edKO mice.

### Par3/aPKCλ promote growth and survival signaling following DMBA exposure

We next asked how loss of aPKCλ and/or Par3 counteract tumor initiation and promotion, and investigated different signaling cascades important for two-stage skin carcinogenesis [24]. Western Blot analysis revealed a similar, significant reduction in ERK1/2 activity, but not levels, in DMBA-treated *Par3*eKO, *aPKCλ*eKO, and edKO mice compared to controls (Fig. 2g–i), which was not observed in non-treated epidermis (Fig. 2j, Suppl.Fig. 2F). Thus, in line with our previous in vitro results [13], aPKCλ and Par3 function in concert to stimulate ERK in vivo under carcinogenesis-inducing conditions.

Akt activation promotes keratinocyte survival in DMBA-induced skin carcinogenesis [25]. Interestingly, loss of Par3, aPKCλ, or both resulted in significantly reduced in vivo Akt activity, but not levels (Fig. 2k–m), which was most prominent in *aPKCλ*eKO or edKO mice, potentially explaining the increased apoptosis in these mice (Fig. 2d). No change in Akt activity and levels was observed in untreated mice irrespective of genotype (Fig. 2n, Suppl.Fig. 2I). Together, these results indicate that the aPKCλ/Par3 complex is essential to induce early growth and survival signaling in response to DMBA.

### Reduced NF-κB and Stat3 activation upon Par3/aPKCλ loss

**Fig. 3:**
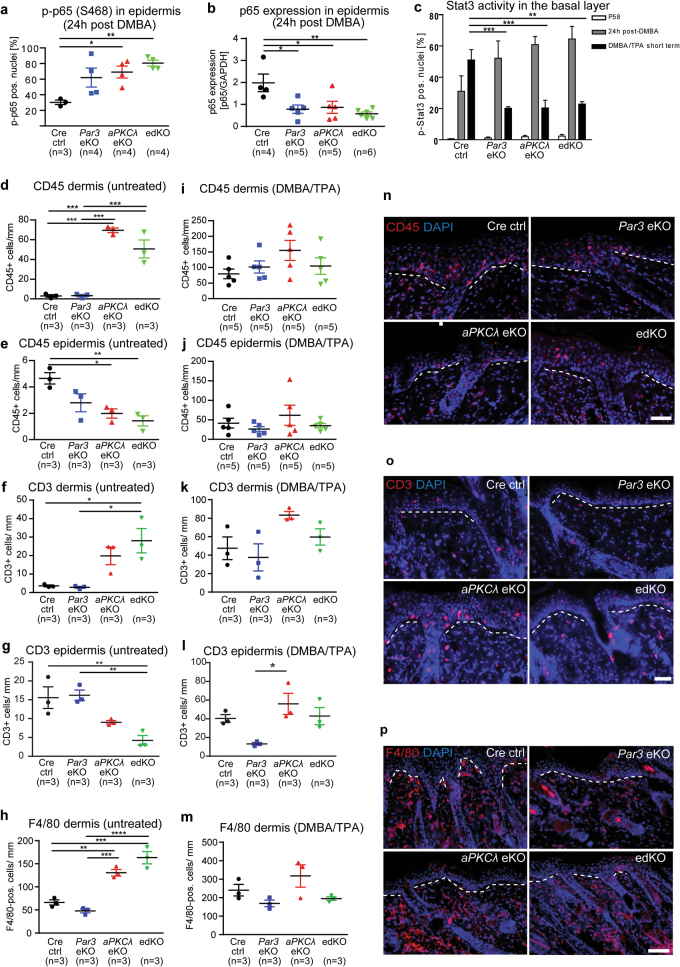
Par3 and aPKCλ control carcinogen-induced inflammatory signaling but not early immune cell recruitment. **a** Quantification of immunofluorescence analysis as previously described (2B) for p-p65 (S468) (Cell Signaling Techn. #3039) on paraffin sections of mice 24 h after DMBA treatment (% of DAPI positive cell nuclei; mean ± SEM; *p < 0.05, **p < 0.01; one-way ANOVA/Tukey’s post-hoc test). **b** Quantification of western blot analysis for total p65 protein in epidermal lysates of mice 24 h after DMBA treatment (mean ± SEM; *p < 0.05, **p < 0.01; one-way ANOVA/Tukey’s post-hoc test). Sample preparation and western blot analysis was performed as previously described [17]. **c** Quantification of immunofluorescence analysis as previously described (2B) for p-STAT3 (Y705) (Cell Signaling Techn. #9145) on paraffin sections of short-term DMBA/TPA treated mice (% of DAPI positive cell nuclei; mean + SEM; **p < 0.01, ***p < 0.001; P58: *n*(*Cre* ctrl) = 5, *n*(*Par3*eKO) = 4, *n*(*aPKCλ*eKO) = 3, *n*(edKO) = 5; 24 h post-DMBA: *n*(*Cre* ctrl) = 3, *n*(*Par3*eKO) = 4, *n*(*aPKCλ*eKO) = 4, *n*(edKO) = 3; short-term DMBA/TPA: *n*(*Cre* ctrl) = 5, *n*(*Par3*eKO) = 5, *n*(*aPKCλ*eKO) = 4, *n*(edKO) = 4; one-way ANOVA/Tukey’s post-hoc). For short-term DMBA/TPA treatments, at P58 mice were treated with a single dose of DMBA (40 μg in 200 μl acetone, Sigma) and three applications of TPA (200 μl of 10^−4^M TPA/acetone) at P65, 68 and 72, and killed 2 days after the last TPA treatment. **d** Quantification of CD45-positive cells/mm dermis of untreated mice as described in 2B (mean ± SEM; ***p < 0.001; one-way ANOVA/Tukey’s post-hoc test). **e** Quantification of CD45-positive cells/ mm epidermis of untreated mice (mean ± SEM; *p < 0.05, **p < 0.01; one-way ANOVA/Tukey’s post-hoc test). **f** Quantification of CD3-positive cells/ mm dermis of untreated mice (mean ± SEM; one-way ANOVA; Tukey’s post-hoc test, *p < 0.05). **g** Quantification of CD3-positive cells/ mm epidermis of untreated mice (mean ± SEM; one-way ANOVA/Tukey’s post-hoc test, *p < 0.05). **h** Quantification of F4/80-positive cells/ mm dermis of untreated mice (mean ± SEM; **p < 0.01; ***p < 0.001; ***p < 0.001; one-way ANOVA; Tukey’s post-hoc test). **i** Quantification of CD45-positive cells/mm dermis of short-term DMBA/TPA treated mice (mean ± SEM; one-way ANOVA/Tukey’s post-hoc test). **j** Quantification of CD45-positive cells/ mm epidermis of short-term DMBA/TPA treated mice (mean ± SEM; one-way ANOVA/Tukey’s post-hoc test). **k** Quantification of CD3-positive cells/mm dermis of short term DMBA/TPA treated mice (mean±SEM, one-way ANOVA/Tukey’s post-hoc test). **l** Quantification of CD3-positive cells/mm epidermis of short-term DMBA/TPA treated mice (mean ± SEM, *p < 0.05, one-way ANOVA/Tukey’s post-hoc test). **m** Quantification of F4/80-positive cells/mm dermis of short term DMBA/TPA-treated mice (mean ± SEM, one-way ANOVA/Tukey’s post-hoc test). **n** Immunofluorescence analysis for CD45 (12-0451-82, Invitrogen) on paraffin sections of short-term DMBA/TPA-treated mice (scale bar = 50 µm). Immunofluorescence analysis was performed as described in 1E. Nuclei were counterstained with DAPI. **o** Immunofluorescence analysis for CD3 (AM11102PU-S, Acris) on paraffin sections of short-term DMBA/TPA-treated mice (scale bar: 50 µm). Immunofluorescence analysis was performed as previously described including additional antigen retrieval prior to blocking via incubation in 3% H_2_O_2_/0.5% KOH (Sigma-Aldrich) for 30 min at 37 °C. Nuclei were counterstained with DAPI. **p** Immunofluorescence analysis for F4/80 (MCA497PET, ABD serotec) as previously described (3D) on paraffin sections of short-term DMBA/TPA-treated mice (scale bar = 100 µm). Nuclei were counterstained with DAPI. See Suppl. Table 1 for antibody dilutions

One hallmark of non-melanoma skin cancer is an inflammatory tumor-promoting micro-environment, faithfully reproduced by the DMBA/TPA skin cancer protocol [21, 22]. Potent regulators of inflammatory signals are NF-κB and Stat3 [26, 27]. Loss of the NF-κB subunit p65 impaired immune cell recruitment during Ras-mediated skin carcinogenesis [28]. Both aPKCλ and Par3 have been implicated in NF-κB signaling even though conflicting data exist whether they exert inhibitory or activating signals controlling NF-κB and immune responses [29, 30]. Interestingly, DMBA-treated *Par3*eKO, *aPKCλ*eKO, and edKO mice displayed an increased number of nuclear pS468-p65-positive cells, indicative of reduced NF-κB signaling [31], as well as reduced total p65 protein levels, albeit only statistically significant for *aPKCλ*eKO and edKO mice, with no significant changes in non-treated skin (Fig. 3a, b, Suppl.Fig. 3A–C).

NF-κB cooperates with Stat3 signaling in different cancers [32]. Constitutive Stat3 activation fosters skin tumor progression [33], whereas its loss impairs skin tumorigenesis [34]. Intriguingly, while Stat3 activity was similar in untreated and 24 h DMBA-treated epidermis (Fig. 3c), it was significantly reduced in all polarity mutant mice subjected to DMBA combined with three subsequent doses of TPA (“short-term DMBA/TPA”) (Fig. 3c, Suppl.Fig. 3D). Thus, aPKCλ and Par3 act in concert in skin carcinogenesis to promote cancer-associated NF-κB and Stat3 signals. Our results agree with observations that depletion of either aPKCλ in prostate cancer cells [35], or of Par3 in lung squamous cell carcinoma cells and ovarian cancer cells impaired Stat3 activity [10, 36], but contrast findings in breast cancer cells, in which Par3 inhibits aPKC and, as consequence, NF-κB/Stat3 activation [4, 5]. Thus, different tumor entities elicit either cooperative or antagonizing Stat3 responses of aPKCλ and Par3. Together, the data strongly suggest that Par3 and aPKCλ not only promote tumor initiation but also TPA-mediated inflammatory responses.

### Distinct role of aPKCλ in controlling skin immune homeostasis, whereas overall DMBA/TPA-induced immune cell infiltration is Par3/aPKCλ-independent

To address whether loss of Par3, aPKCλ, or both would affect immune cell homeostasis already in untreated skin, we performed immunohistochemical staining for CD45 (pan-leukocyte marker), CD3 (T-cells) and F4/80 (myeloid cells, mostly macrophages). Interestingly, immunofluorescence analysis revealed a strongly increased number of CD45^+^ cells (Fig. 3d, Suppl.Fig. 3F), CD3^+^ cells (Fig. 3f) and F4/80^+^ cells (Fig. 3h) in the dermis of untreated P58 *aPKCλ*eKO and edKO mice but not in control or *Par3*eKO mice. In contrast, in the epidermis immune cell numbers and, more specifically, CD3^+^ T-cell numbers, were reduced upon loss of aPKCλ or both aPKCλ/Par3 (Fig. 3e, g). These data indicate a Par3-independent role for aPKCλ in immune cell recruitment at homeostatic conditions. Short-term DMBA/TPA treatment resulted in similar numbers of dermal and largely similar epidermal immune cells (CD45^+^, Fig. 3i, j, n), and more specifically T-cells (Fig. 3k, l, o) or macrophages (Fig. 3m, p) in all genotypes. Moreover, staining for markers that suggest tumor-promoting (IL4R) or tumor-suppressive (iNOS) macrophage identity [37] did not reveal any consistent changes either (data not shown). Thus, despite reduced epidermal inflammatory signals, and in contrast to epidermal *p65*eKO mice [28], the reduced tumor burden in *Par3*/*aPKCλ*-single or double-mutant mice cannot directly be attributed to an early reduction in infiltrating immune cells.

### Par3 and aPKCλ control sustained DMBA/TPA-induced proliferation and survival

**Fig. 4:**
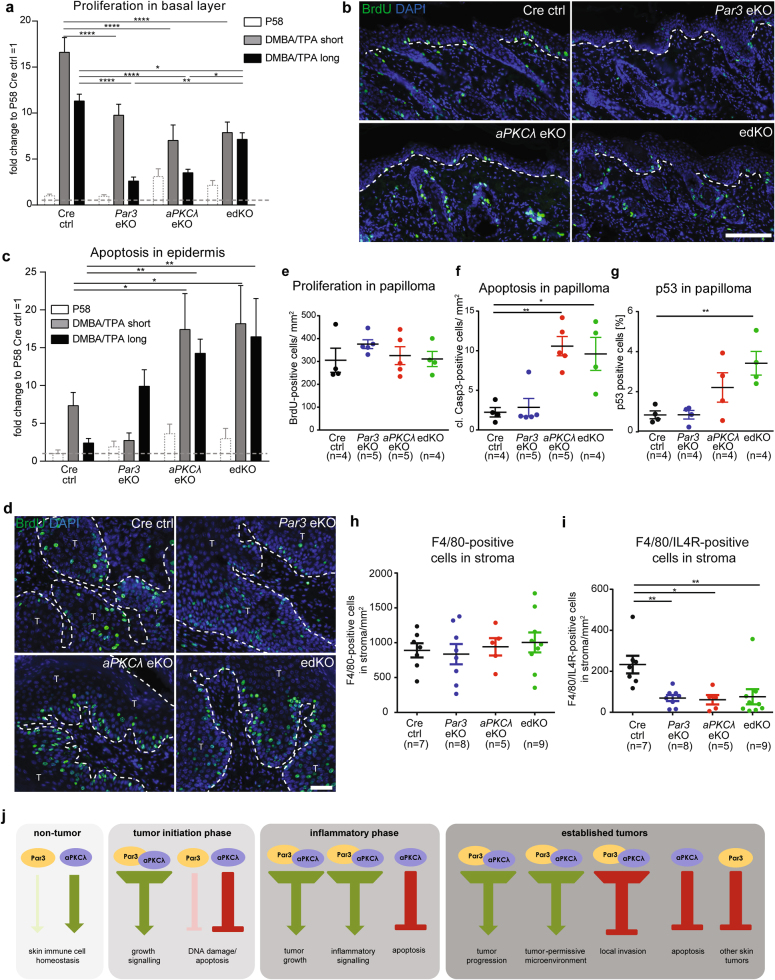
Par3 and aPKCλ control DMBA/TPA-induced early hyperproliferation and subsequent macrophage phenotypes in established tumors. **a** Quantification of BrdU-positive cell nuclei upon short- and long-term DMBA/TPA treatment and in untreated mice at P58 (% of DAPI positive cell nuclei; fold change to untreated Cre ctrl = 1; mean + SEM; *n* > 3/ genotype; ****p < 0.0001; **p < 0.01; *p < 0.05; two-way ANOVA/Tukey’s post-hoc test). **b** Immunofluorescence analysis as described in Fig. 2a for BrdU (#347580, BD Bioscience) on paraffin sections of mice after long-term DMBA/TPA treatment (scale bar = 100 µm). Nuclei were counterstained with DAPI. **c** Quantification of cleaved Caspase 3-positive cell nuclei upon short- and long-term DMBA/ TPA treatment and in untreated mice at P58 (% of DAPI-positive cell nuclei; fold change to untreated Cre ctrl = 1; mean + SEM; *n* > 3/genotype; **p < 0.01; *p < 0.05; two-way ANOVA/Dunnett’s post-hoc test). **d** Immunofluorescence analysis as previously described (2A) for BrdU (#347580, BD Bioscience) on paraffin sections of papilloma of mice after long-term DMBA/TPA treatment (scale bar = 50 µm). Nuclei were counterstained with DAPI. **e** Quantification of BrdU-positive cell nuclei in papilloma after long-term DMBA/TPA treatment (BrdU-positive cells/ mm^2^ tumor; mean ± SEM, one-way ANOVA/Dunnett’s post-hoc test). **f** Quantification of cleaved Caspase 3 (cl.Casp3)-positive cell nuclei in papilloma after long-term DMBA/TPA treatment (cl. Casp3-positive cells/mm^2^ tumor; mean ± SEM; **p < 0.01, one-way ANOVA/Dunnett’s post-hoc test). **g** Quantification of p53-positive cell nuclei in papilloma after long-term DMBA/TPA treatment (% of DAPI-positive cell nuclei; *n* = 4 mice/genotype; mean ± SEM; **p < 0.01; one-way ANOVA/Dunnett’s post hoc test). **h** Quantification of F4/80-positive cells per mm^2^ in stroma of papillomas of long-term DMBA/TPA-treated mice (mean ± SEM, one-way ANOVA/Tukey’s post-hoc test). **i** Quantification of F4/80/IL-4Rα-double-positive cells per mm^2^ in stroma of papillomas of long-term DMBA/TPA-treated mice (mean ± SEM, **p < 0.01, *p < 0.05, one-way ANOVA/Tukey’s post-hoc test). **j** Overview scheme summarizing the findings of this study. See Suppl. Table 1 for antibody dilutions

We next asked whether aPKCλ and Par3 cooperate in the outgrowth of *Hras*-mutated keratinocytes. As expected, short-term DMBA/TPA treatment strongly induced proliferation in control mice as measured by BrdU incorporation. This induction was significantly reduced in either *Par3*eKO or *aPKCλ*eKO mice, with no further decrease in edKO mice (Fig. 4a), in agreement with the lowered ERK activity induced by DMBA (Fig. 2g, h). Moreover, even though upon long-term DMBA/TPA treatment ERK, Akt and NF-κB signaling were similar (Suppl.Fig.2G,H,J,K, Suppl.Fig.3E), proliferation remained significantly reduced in all *Par3/aPKCλ*-mutant mice (Fig. 4a, b), albeit that single *Par3*eKO and *aPKCλ*eKO mice showed a significant stronger reduction than edKO mice. In conclusion, aPKCλ/Par3 cooperate to induce early and sustain long-term hyperproliferation in chemical skin carcinogenesis.

We next examined how loss of Par3 or aPKCλ affects apoptotic responses in early vs. late tumor promotion stages. Short-term DMBA/TPA treatment did not significantly alter apoptosis in *Par3*eKO mice, whereas apoptosis was increased in *aPKCλeKO* and edKO mice (Fig. 4c), similar to DMBA treatment alone (Fig. 2c, d), and to *aPKCλ*-KO keratinocytes transfected with oncogenic HRasV12 and exposed to TPA (Suppl.Videos 1, 2; Suppl.Fig. 4B). Long-term DMBA/TPA treatment also elicited an increase in cleaved Caspase3-positive cells in *aPKCλ*eKO and edKO mice, whilst a trend to increased apoptosis was observed in *Par3*eKO mice (Fig. 4c), pointing again to a differential requirement and/or timing of aPKCλ and Par3 in counteracting cell death at different stages of tumor formation and progression.

### Reduced tumor growth correlates with increased apoptosis and macrophage subtype

*Par3*eKO, *aPKCλ*eKO, and edKO mice displayed significantly decreased tumor sizes compared to controls (Fig. 1b). We thus asked whether this reduction is due to reduced tumor cell proliferation. Unlike earlier tumorigenesis stages, however, BrdU incorporation, ERK, Akt, and NF-κB activities were largely comparable between tumor tissues of all experimental groups (Fig. 4d, e, Suppl.Fig. 4D-I), suggesting that tumors—once established—can bypass aPKCλ/Par3-dependent growth signals, possibly through accumulating additional mutations [22, 23]. In contrast, apoptotic cell numbers were significantly increased particularly in aPKCλ-deficient papillomas (Fig. 4f). In agreement, only loss of aPKCλ but not Par3 increased the number of p53-positive tumor cells (Fig. 4g), potentially explaining a more pronounced reduction in tumor size in *aPKCλ*eKO as compared to *Par3*eKO mice (Fig. 1b). Together, the results identify aPKCλ as major regulator of cell survival during DMBA-induced skin carcinogenesis. Of note, although Par3 or aPKC mislocalization has been associated with altered function in different cancers [10–14], and in skin tumors Par3 loss led to cytoplasmic redistribution of aPKC [13], aPKCλ did not seem to control Par3 recruitment (Suppl.Fig. 4A, C).

Finally, we asked whether Par3 and/or aPKCλ controls distinct tumor macrophage subsets that either promote or suppress tumorigenesis [37]. Whereas no change in total F4/80-positive (Fig. 4h) or F4/80/iNOS-double positive macrophages (not shown) in the tumor stroma was detected, the number of F4/80/IL4Rα-double-positive stromal macrophages was significantly reduced in all *aPKCλ/Par3*-mutant mice compared to control (Fig. 4i), suggesting that epidermal aPKCλ and Par3 next to intrinsic oncogenic signaling also promote a tumor-permissive micro-environment, which may contribute to tumor size.

Collectively, this study provides in vivo evidence that Par3 and aPKCλ not only act in concert but also serve independent functions in skin homeostasis and tumorigenesis. Our data genetically demonstrate that Par3 and aPKCλ act in concert (Fig. 4j) to: (1) promote early and sustained tumor outgrowth but inhibit invasion; (2) activate ERK signaling early during tumorigenesis, thus providing in vivo evidence for our previous *in vitro* observations showing that Par3 promotes oncogenic Ras/ERK signaling through aPKC [13]; (3) enhance epidermal Akt, NF-κB, and Stat3 signaling; (4) foster a tumor-permissive micro-environment, which taken together explain how aPKC and Par3 cooperate to (5) promote non-melanoma skin tumor incidence and burden. Interestingly, despite early changes in epidermal inflammatory signals no changes were observed in immune cell recruitment, either at early or late stages of carcinogenesis, suggesting an epidermal tumor-intrinsic role for NF-κB/Stat3, e.g., in tumor outgrowth and DNA damage responses, in addition to promoting a pro-tumorigenic micro-environment for tumors. Importantly, our data also provide potential evidence for a Par3-independent role for aPKCλ in immune cell homeostasis as well as cell survival at different stages of tumorigenesis, whereas Par3 serves a tumor-suppressive function not shared by aPKCλ (Fig. 4j). How aPKCλ and Par3 control these functions will be an important future goal.

Taken together, our data show that Par3 and aPKCλ act in concert to promote different stages of skin tumor initiation and progression. Based on the many biochemical data, they likely do so as a complex through fostering pro-proliferative, and tumor-promoting pro-inflammatory signals in epithelia executed by ERK, Akt, NF-κB, and Stat3.

## Electronic supplementary material

Inventory Supplementary Material

Supplementary Figure 1

Supplementary Figure 2

Supplementary Figure 3

Supplementary Figure 4

Supplementary Table 1

Legends to Supplementary Videos

Supplementary Video 1

Supplementary Video 2
