# Supplementary figures and images for: Shared and independent functions of aPKCλ and Par3 in skin tumorigenesis
Source: Oncogene. 2018 May 23;37(37):5136–46. doi: 10.1038/s41388-018-0313-1 (PMC6137026; doi:10.1038/s41388-018-0313-1)

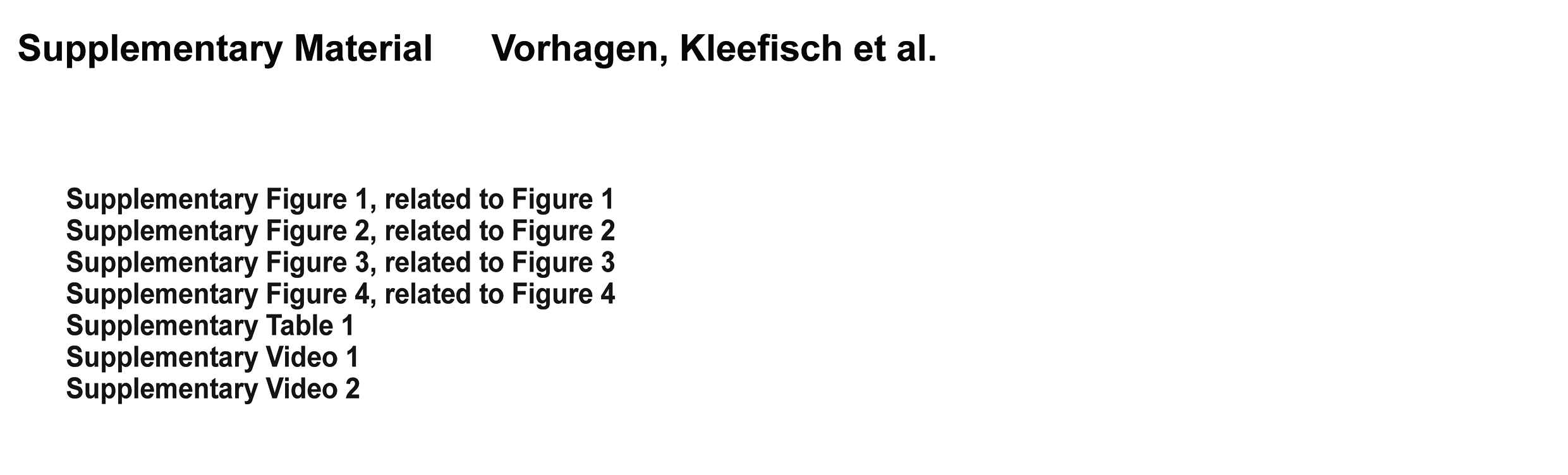

Supplement: Supplementary file 1 — Inventory Supplementary Material [file 41388_2018_313_MOESM1_ESM.tif]

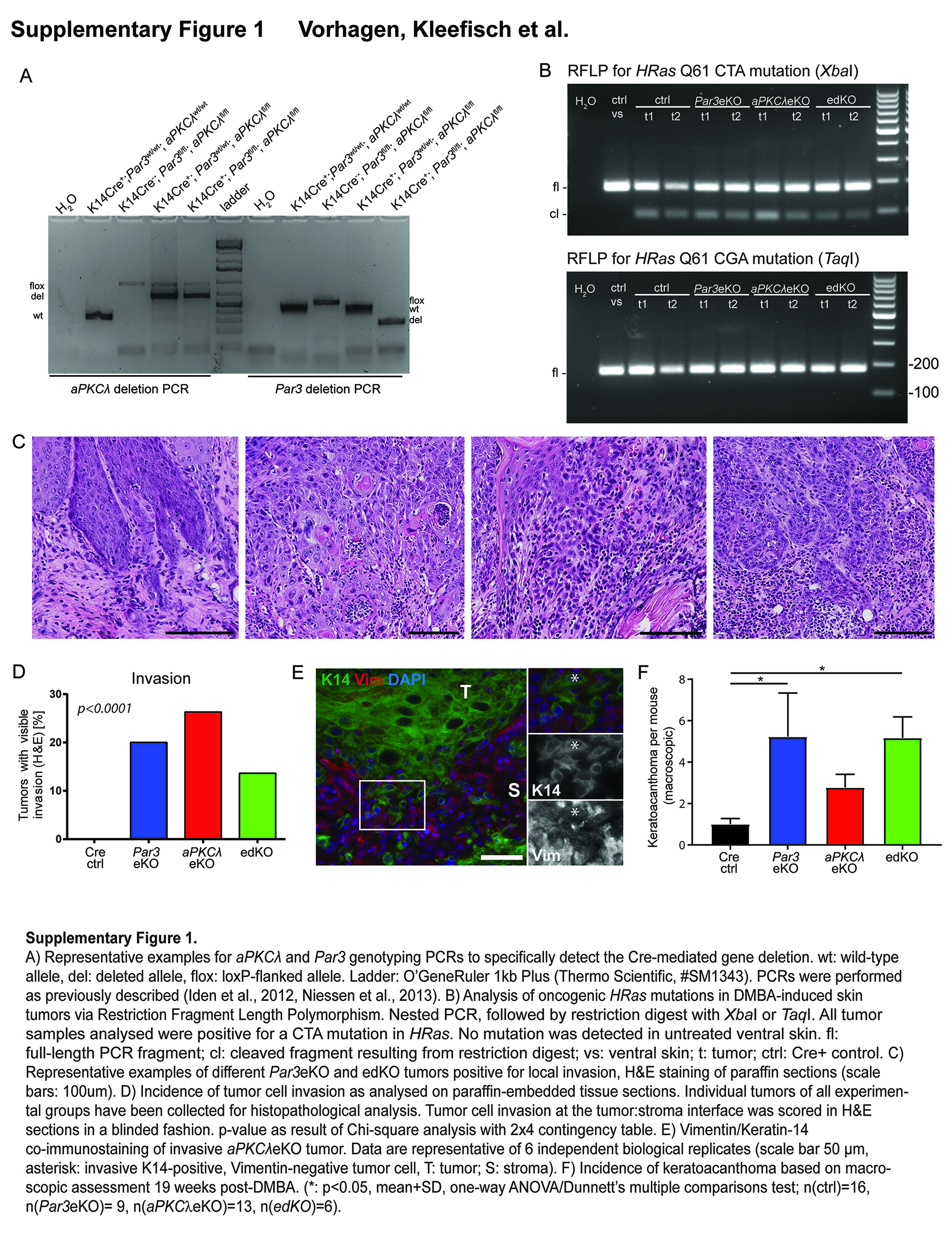

Supplement: Supplementary file 2 — Supplementary Figure 1 [file 41388_2018_313_MOESM2_ESM.tif]

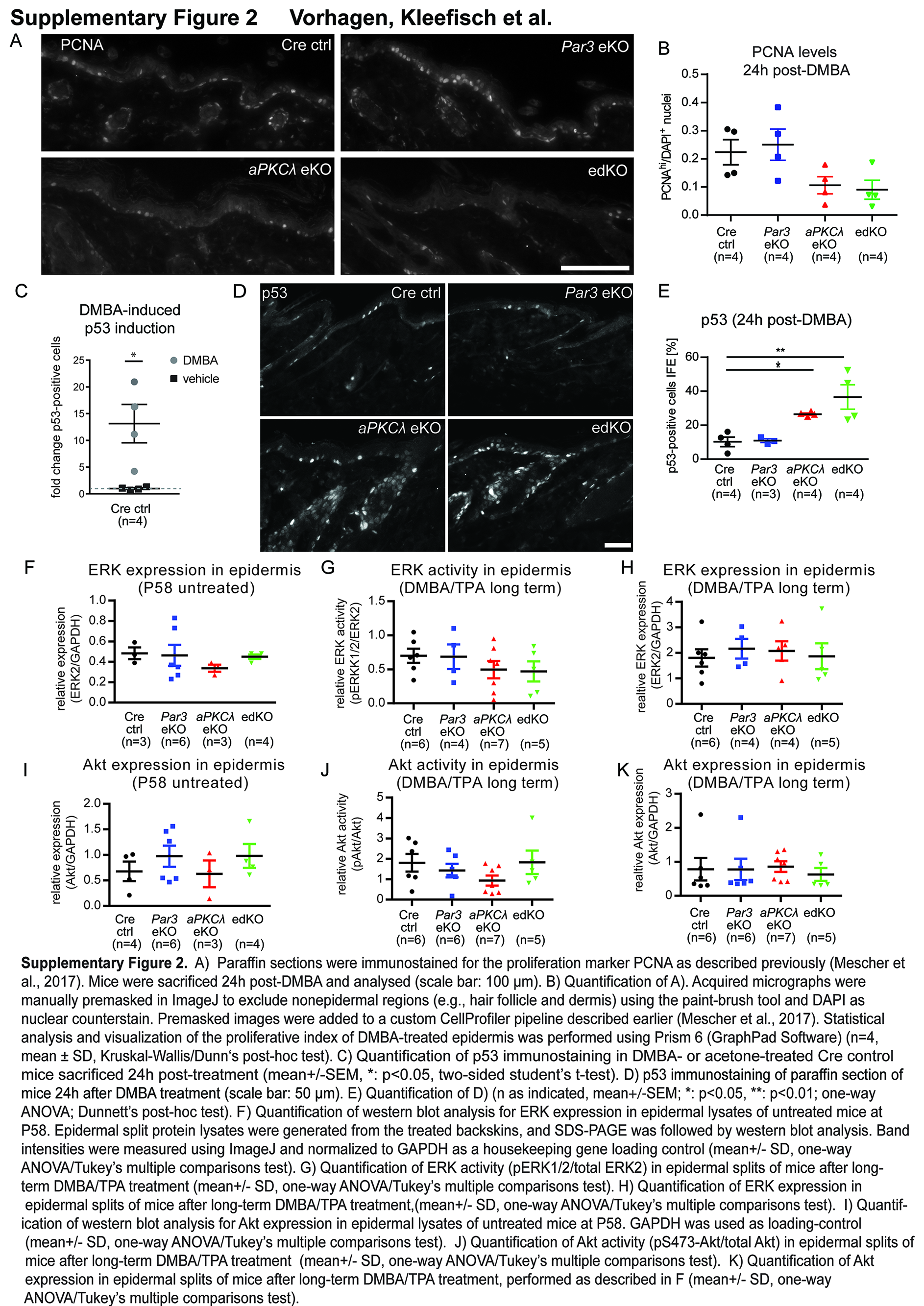

Supplement: Supplementary file 3 — Supplementary Figure 2 [file 41388_2018_313_MOESM3_ESM.tif]

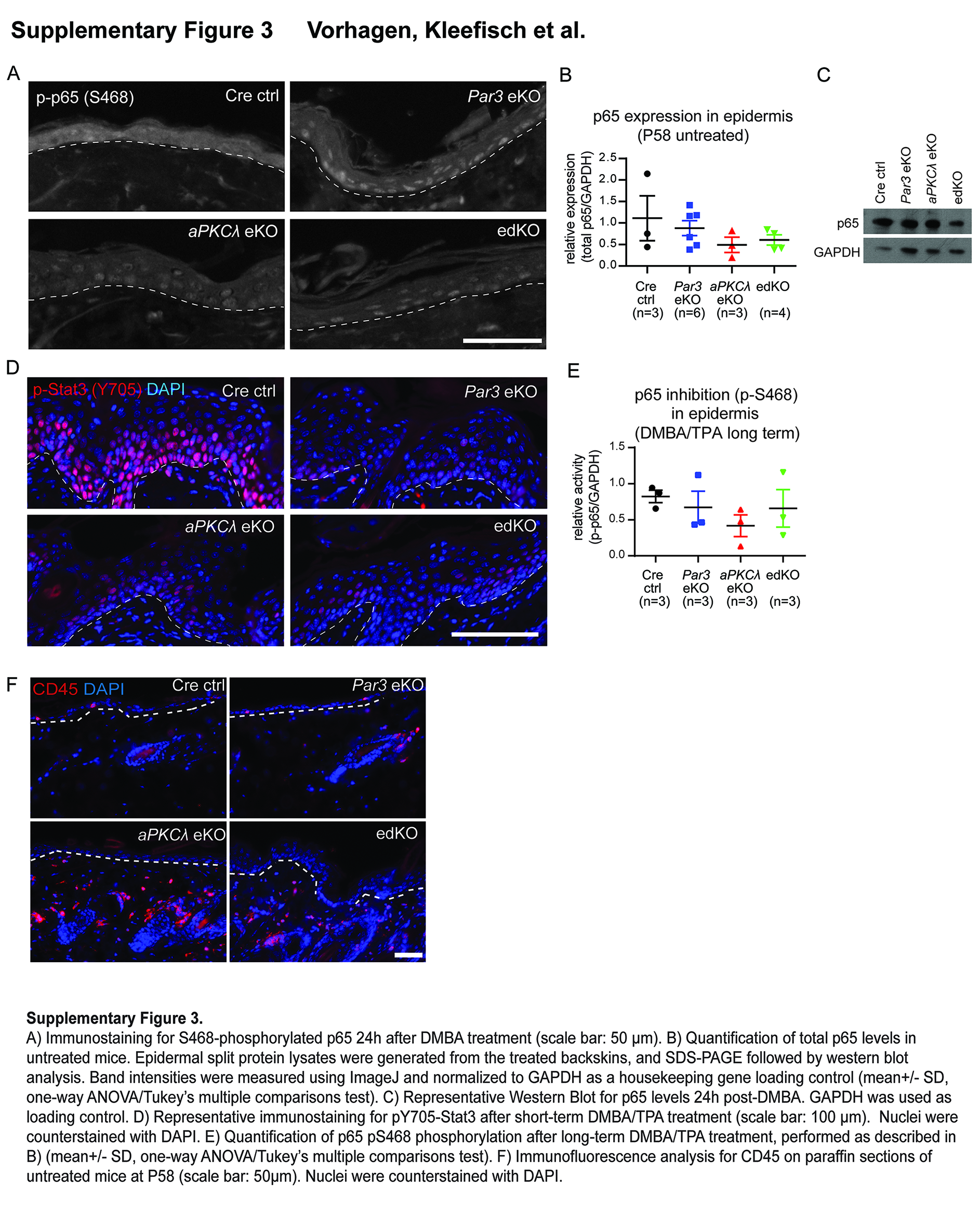

Supplement: Supplementary file 4 — Supplementary Figure 3 [file 41388_2018_313_MOESM4_ESM.tif]

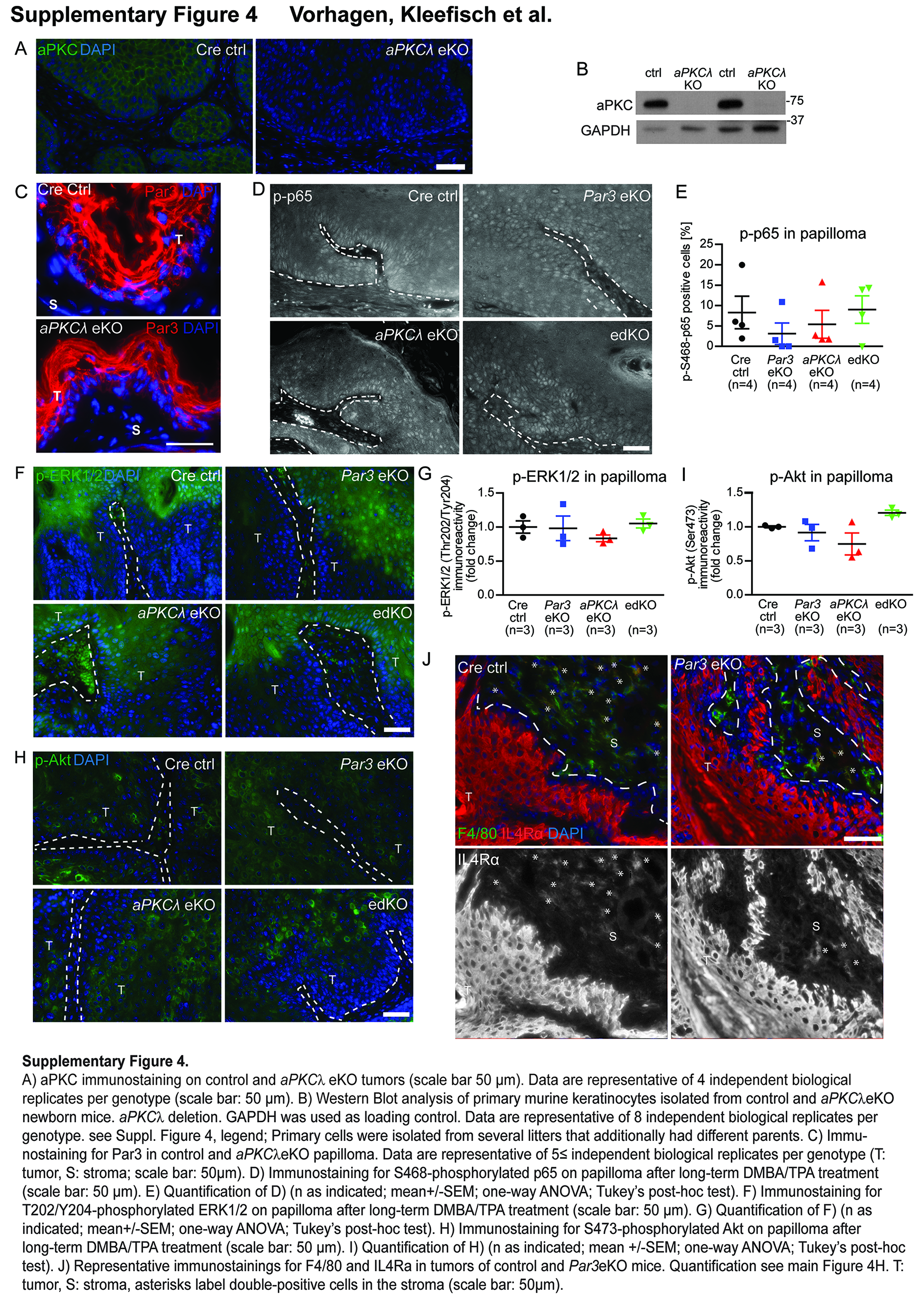

Supplement: Supplementary file 5 — Supplementary Figure 4 [file 41388_2018_313_MOESM5_ESM.tif]

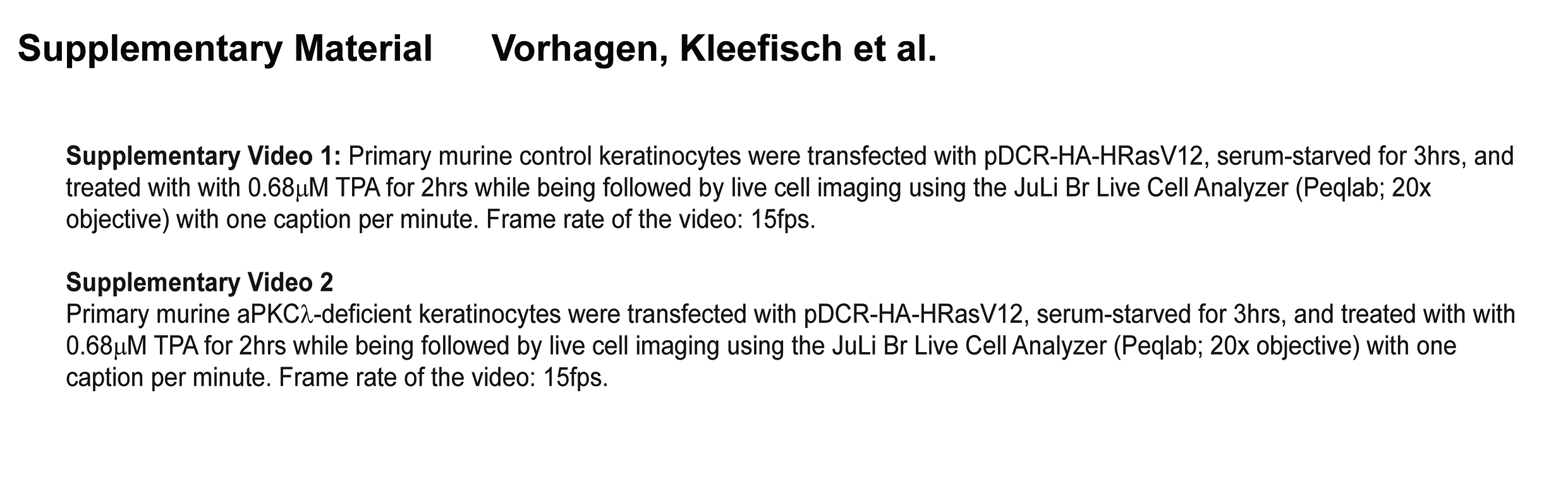

Supplement: Supplementary file 7 — Legends to Supplementary Videos [file 41388_2018_313_MOESM7_ESM.tif]
