# Supplementary material for: Shared and independent functions of aPKCλ and Par3 in skin tumorigenesis
Source: Oncogene. 2018 May 23;37(37):5136–46. doi: 10.1038/s41388-018-0313-1 (PMC6137026; doi:10.1038/s41388-018-0313-1)
Supplement: Supplementary file 6 — Supplementary Table 1 [file 41388_2018_313_MOESM6_ESM.docx]

**Supplementary Table 1 Vorhagen, Kleefisch et al.**

| **Primary antibodies** | **Catalog number** | **Species** | **Dilution** |
| --- | --- | --- | --- |
| Akt (Cell Signaling) | 9272 | rabbit | WB – 1:1000 |
| aPKC ζ (C-20) (Santa Cruz) | sc-216 | rabbit | IF – 1:200 |
| aPKC ζ (H-1) (Santa Cruz) | sc-17781 | mouse | WB – 1:500 |
| BrdU (DAKO Cytomation) | MO744 | mouse | IF – 1:50 |
| CD3 (Acris) | AM11102PU-S | rabbit | IF – 1:200 |
| CD45 (30-F11) PE (Invitrogen) | 12-0451-82 | rat | IF – 1:300 |
| cleaved Caspase 3 (R&D) | AF835 | rabbit | IF – 1:500 |
| cleaved Caspase 3 (R&D) | Mab835 | rabbit | WB – 1:1000 |
| ERK2 (BD Transduction) | 610103 | mouse | WB – 1:5000 |
| F4/80 (ABD serotec) | MCA497PET | rat IgG2b | IF – 1:100 |
| GAPDH (Millipore) | MAB374 | mouse | WB – 1:18.000 |
| Il-4Ralpha (Santa Cruz) | sc-686 | rabbit | IF – 1:100 |
| Keratin14 (Covance) | PRB-155P | rabbit | IF – 1:1000 |
| p53 (Leica) | NCL-L-p53-CM5p | rabbit | IF – 1:200 |
| p65 (Cell Signaling) | 8242 | rabbit | WB – 1:1000 |
| Par3 (Millipore) | 07-330 | rabbit | IF – 1: 400 |
| PCNA (Calbiochem) | NA03 | mouse | IF – 1:100 |
| phospho-Akt (S473) XP (Cell Signaling) | 4060 | rabbit | WB – 1:1000 |
| phospho-Akt (Ser473) (Cell signaling) | 9277 | rabbit | IF – 1:200 |
| phospho-ERK1/2 XP (T202/Y204) (Cell Signaling) | 4370 | rabbit | WB – 1:1000  IF – 1:200 |
| phospho-p65 (S468) (Cell Signaling) | 3039 | rabbit | IF – 1:500  WB – 1:1000 |
| phospho-Stat3 (Y705) (Cell Signaling) | 9145 | rabbit | IF – 1:500 |
| Stat3 (Santa Cruz) | sc483 | rabbit | WB – 1:500 |
| Vimentin (Fitzgerald) | 20R-VP004 | guinea pig | IF – 1:200 |
| γH2AX; S139 (Cell Signalling) | 9718 | rabbit | IF – 1:500 |
| **Secondary antibodies for immunofluorescence analyses** | | | |
| AlexaFluor 488 α-mouse (Invitrogen) | A21202 | donkey | IF – 1:500 |
| AlexaFluor 568 α-rabbit (Invitrogen) | A11036 | goat | IF – 1:500 |
| AlexaFluor 568 α-rat (Invitrogen) | A11077 | goat | IF – 1:500 |
| AlexaFluor 594 anti-mouse (Invitrogen) | A21203 | donkey | IF – 1:500 |
| AlexaFluor 488 α-rabbit (Invitrogen) | A21206 | donkey | IF – 1:500 |
| AlexaFluor 594 α-rabbit (Invitrogen) | A21207 | donkey | IF – 1:500 |
| AlexaFluor 594 α-guinea pig (Invitrogen) | A11076 | goat | IF – 1:500 |
| **Secondary antibodies for western blot analyses** | | | |
| HRP α-rabbit (GE Healthcare) | NA9340V | donkey | WB – 1:4000 |
| HRP α-mouse (GE Healthcare) | NA931V | sheep | WB – 1:4000 |
